# Fitting and Calibrating a Multilevel Mixed-Effects Stem Taper Model for Maritime Pine in NW Spain

**DOI:** 10.1371/journal.pone.0143521

**Published:** 2015-12-02

**Authors:** Manuel Arias-Rodil, Fernando Castedo-Dorado, Asunción Cámara-Obregón, Ulises Diéguez-Aranda

**Affiliations:** 1 Unidad de Gestión Forestal Sostenible (UXFS), Departamento de Ingeniería Agroforestal, Universidade de Santiago de Compostela, Escuela Politécnica Superior, C/Benigno Ledo, Campus universitario, 27002 Lugo, Spain; 2 Departamento de Ingeniería y Ciencias Agrarias, Universidad de León, Escuela Superior y Técnica de Ingeniería Agraria, Avda de Astorga, 24400 Ponferrada, Spain; 3 Grupo de Investigación en Sistemas Forestales Atlánticos (GIS-Forest), Departamento de Biología de Organismos y Sistemas, Universidad de Oviedo, Escuela Politécnia de Mieres, C/Gonzalo Gutiérrez Quirós s/n, 33600 Mieres, Spain; University of Vigo, SPAIN

## Abstract

Stem taper data are usually hierarchical (several measurements per tree, and several trees per plot), making application of a multilevel mixed-effects modelling approach essential. However, correlation between trees in the same plot/stand has often been ignored in previous studies. Fitting and calibration of a variable-exponent stem taper function were conducted using data from 420 trees felled in even-aged maritime pine (*Pinus pinaster* Ait.) stands in NW Spain. In the fitting step, the tree level explained much more variability than the plot level, and therefore calibration at plot level was omitted. Several stem heights were evaluated for measurement of the additional diameter needed for calibration at tree level. Calibration with an additional diameter measured at between 40 and 60% of total tree height showed the greatest improvement in volume and diameter predictions. If additional diameter measurement is not available, the fixed-effects model fitted by the ordinary least squares technique should be used. Finally, we also evaluated how the expansion of parameters with random effects affects the stem taper prediction, as we consider this a key question when applying the mixed-effects modelling approach to taper equations. The results showed that correlation between random effects should be taken into account when assessing the influence of random effects in stem taper prediction.

## Introduction

Maritime pine (*Pinus pinaster* Ait.) is the most common forest species in Spain (15%), where it currently yields 27% of the annual timber volume harvested [1]. Timber produced by this species is usually destined for sawing, pulp and wood-based panels, depending on the log dimensions [2]. Maritime pine is well-represented in the region of Asturias (NW Spain), where it occupies more than 22,000 ha (almost 5% of the forest area) [3].

Accurate volume predictions to any merchantable limit have always been a matter of interest to forest managers. Several methods can be used to estimate merchantable volume, although the most common is integration of a taper function along the bole (e.g. [4–6]). According to [7], together with total and merchantable volume, taper functions can also provide forest managers with estimates of the following: (i) diameter at any point along the stem, (ii) merchantable height to any top diameter, and (iii) individual log volumes of any length at any height from the ground.

Taper functions are usually classified as single, segmented and variable-exponent ([8, 9], p. 12). Although many taper functions have been reported in the forestry literature over the past few decades, there seems to be general agreement that none of them performs particularly well across multiple species (e.g. [10]). Nevertheless, variable-exponent models usually provide the most accurate predictions [11–13]. These models consist of a continuous function for describing the shape of the bole by using a changing exponent to describe the lower (neiloid), mid (paraboloid) and upper (conic) forms of the stem.

When fitting taper functions, the structure of the dataset is usually grouped, i.e. longitudinal measurements within the same tree (diameters along the stem) are grouped into an upper hierarchy at plot level (several trees per plot). As a consequence, within-tree observations are likely to be correlated, which implies that the significance of parameter estimates obtained by the ordinary least squares (OLS) technique is not reliable ([14], p. 288). Mixed-effects models allow the autocorrelation to be at least partly accounted for, by the inclusion of random effects ([9], pp. 36–41). This approach also enables the variability between trees within the same plot and the variability between plots (i.e. two grouping factors) to be accounted for. This can be useful for calibrating (also known as localizing) the model for a specific subject if at least one additional measurement from this subject is available [15]. To our knowledge, multilevel mixed-effects models have been applied in several studies within the forestry modelling framework [16–19], although they have not previously been considered for nonlinear stem taper functions. This represents an important advance in relation to other studies based on the application of the mixed-effects modelling approach to nonlinear stem taper functions (e.g. [13, 15, 20]), in which the correlation between trees of the same plot has been ignored. The approach also enables researchers to determine how the random variation in stem shape is split into portions attributable to different hierarchical levels (plot and tree in this case, [17]).

In mixed-effects taper modelling, it is essential to understand how the expansion of parameters with random effects affects stem taper prediction. [12] evaluated how the stem taper varies with individual variations in the values of several parameters of the taper function. Nevertheless, this approach (op. cit.) may be not reliable when more than one parameter is expanded with random effects, as it does not take into account correlation between parameters. To our knowledge, this correlation has not been previously considered in assessing the sensitivity of the stem taper to changes in model parameters.

The objectives of the present study were as follows: (i) to fit a stem taper function for *Pinus pinaster* Ait. in Asturias by using a multilevel mixed-effects modelling approach; (ii) to evaluate the proportion of variability explained at plot and tree levels; (iii) to select the best combination of parameters to expand with random effects; (iv) to recommend the best stem location to measure an additional diameter for calibrating the mixed-effects model; and (v) to assess the sensitivity of stem taper prediction to parameter variations.

## Materials and Methods

The data used in this study were obtained from a network of 73 research plots established in 2007 in pure even-aged maritime pine stands. The plots were installed in forests privately owned by local communities but managed by the regional government of Asturias, which was the institution that allowed the plot establishment through the technicians of the Forest Service. They covered the distribution area of this species in Asturias, comprising the existing range of ages, stand densities and site qualities. Taper data were measured in six trees (two dominant, two intermediate, and two suppressed) located close to each plot (except in one plot where only two trees were felled). Diameter at breast height (*d*, cm) was measured to the nearest 0.1 cm. After felling each tree, total bole length was measured to the nearest 0.01 m to calculate the total tree height (*h*, m). The stem was subsequently cut into logs of 0.3 to 2.5 m length. Diameter over bark (*d*_*i*_, cm) was measured twice, to the nearest 0.1 cm, in each crosscut section, with the second measurement made at right angles to the first. The average diameter was then calculated for each section. The height of each section from ground level (*h*_*i*_, m) was also recorded, to the nearest 0.01 m. Log volumes were calculated as conical frustums, while the top section was treated as a cone. These values were used to obtain the total outside-bark stem volume (*v*, m^3^).

**Fig 1:**
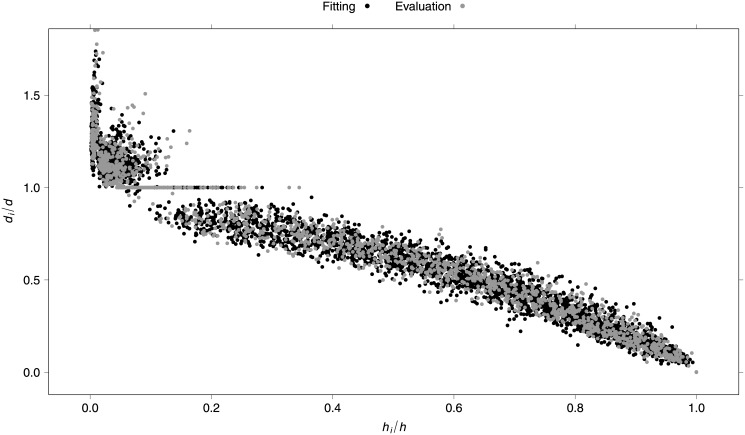
Relative diameter against relative height of fitting and evaluation data sets.

Visual inspection of each stem taper and of a scatter plot of relative diameter (*d*_*i*_/*d*) against relative height (*h*_*i*_/*h*) was used to detect possible outliers. Four trees with abnormal stem taper and extreme data points (caused by large knots or abiotic damage in the stem) were removed from the data set. Subsequently, all plots with fewer than six trees were removed to yield the same number of trees per plot. A total of 5,744 bole sections from 420 trees of 70 plots were finally used for the analysis (S1 Dataset). This data set was then randomly divided by plot: 70% for model fitting and the remaining 30% for evaluation. The summary statistics for both fitting and evaluation data sets are presented in Table 1. Fig 1 shows a scatter plot of relative diameter (*d*_*i*_/*d*) against relative height (*h*_*i*_/*h*) of the final data used, for both fitting and evaluation data sets.

**Table 1:** Data summary.

Variable	Fitting (294 trees)				Evaluation (126 trees)			
	Mean	Min	Max	Std. dev.	Mean	Min	Max	Std. dev.
No. sections	14	7	20	3	14	7	24	3
*d*	23.5	7.15	56.9	10.2	23.4	6.50	52.4	10.1
*h*	13.9	4.58	27.6	5.46	14.1	3.77	29.5	5.82
*h*_*st*_	0.08	0.03	0.40	0.05	0.09	0.03	0.67	0.06
*v*	0.38	0.01	2.67	0.45	0.39	0.01	2.39	0.48

*d*, diameter at breast height (cm); *h*, total tree height (m); *h*_*st*_, stump height (m); *v*, total outside-bark stem volume (m^3^).

### Taper function

The variable-exponent model proposed by [7] (Eq 1) was selected for analysis because it performed best in describing the stem taper of maritime pine in the nearby region of Galicia [11]. Moreover, a number of successful applications of this function involving different species and stem tapers have been reported (e.g. [8, 21, 22]). The expression of this model is as follows:

$$\begin{array}{c} d_{i}=a_{0}d^{a_{1}}h^{a_{2}}x^{\left(b_{1}q^{4}+b_{2}\left(1/\exp\left(d/h\right)\right)+b_{3}x^{0.1}+b_{4}\left(1/d\right)+b_{5}h^{w}+b_{6}x\right)} \end{array}$$ (1)

where $x=\frac{w}{1-{\left(1.3/h\right)}^{\left(1/3\right)}}$, *w* = 1−*q* ^1/3^, *q* = *h*_*i*_/*h*, and *a*_0_−*a*_2_ and *b*_1_−*b*_6_ are parameters.

Some authors (e.g. [12, 23]) have stated that taper functions should be fitted for $d_{i}^{2}$ rather than *d*_*i*_ because the former provides less biased estimates of total or merchantable volumes. Nevertheless, we considered *d*_*i*_ as the dependent variable because estimation of *h*_*i*_ is a key step in classifying stem portions by top diameter limits and log lengths according to the requirements of different industrial destinations (peeling, sawing, pulp wood, etc.). Because the model of [7] has no generalized inverse, the total volumes were computed by numerical integration, using the integrate function of R [24].

### Mixed-effects modelling

The parameter vector of mixed-effects models is allowed to vary between subjects, considering a fixed part (common to the population, fixed-effects parameters, hereafter fixed parameters) and a random component (specific for each subject, i.e. random effects). The random effects are assumed to follow a multivariate normal distribution with mean zero and a positive-definite variance-covariance matrix **D**. A more detailed explanation of mixed-effects modelling can be found in [15, 25, 26], the last two within a taper modelling framework.

A key question in mixed-effects modelling is the definition of which parameters should be considered as fixed and which as mixed (composed of fixed parameters and random effects) ([25], p. 282). Three possible approaches can be used to deal with this task: (i) fitting each individual separately without random effects and selecting the most variable parameters to be expanded with random effects [27], (ii) assessing how the stem taper varies with individual variations in the values of several parameters of the taper function [12], and (iii) evaluating several combinations of parameters to be expanded with random effects. The former presents two problems: (a) individual fitting was not possible in this case as there were not enough observations per tree (average of 14 observations) to obtain significant parameter estimates (Eq 1 has 9 parameters), and (b) high variability of parameters across individuals may not be related to high variability in stem shape prediction, as parameters enter the function in a nonlinear fashion. Concerning the second approach, we demonstrate below that this may not be a good solution in some cases. Therefore, in the present study we used the third approach by considering all possible expansion combinations of one and two parameters (i.e. 9 candidate models with one parameter expanded plus 36 candidate models with two parameters expanded), which yielded 45 candidate mixed-effects models.

As can be inferred from the data description, the data set has a hierarchical structure, with three levels: each plot contains six trees belonging to three sociological classes, and each tree includes several diameter measurements along the stem. Preliminary analysis (results not shown) did not show any differences between the stem taper of different sociological classes, and therefore these were not included in the model as dummy variables. We therefore considered a two-level mixed-effects model, which is able to distinguish among variability between plots and variability between trees within the same plot. The plot level has not been considered in other previous studies (e.g. [13, 15, 20, 28]), and the authors have thus assumed that the stem shape of trees from the same plot are not correlated, which does not seem reasonable [16, 29].

The error term of a mixed-effects model is assumed to follow a multivariate normal distribution with mean zero and a positive-definite variance-covariance matrix (**R**_*i*_). Because the data used consisted of repeated measurements along tree stems, correlations between residuals for the same tree are expected. When mixed-effects modelling did not completely account for this autocorrelation, we modelled the error term with a continuous autoregressive structure (CAR(1)), because the within-tree observations were not equally spaced. Apart from the autocorrelation, the residuals are assumed to be homoscedastic (i.e. independent from predictions and covariates).

Application of mixed-effects modelling involves three steps [30]: (i) estimating model parameters, (ii) predicting random effects, and (iii) making subject-specific (SS) predictions. The first step corresponds to the fitting phase, while the second and third are known as calibration and SS prediction respectively.

### Fitting mixed-effects models

We fitted a nonlinear model linearized by a first-order Taylor series expansion around the random effects ([25], p. 312). Two expansion methods are available: (i) the first-order (FO) approximation of [31] and (ii) the first-order conditional expectation (FOCE) approximation of [32], in which random effects are set to the expected value of zero or to the current estimated best linear unbiased predictor (EBLUP) respectively. The FOCE method provides slightly better results than the FO method, although it fails more often to reach convergence [33]. Within the forestry modelling, [30, 34] found that models fitted by the FO method may yield biologically unreasonable results when making SS predictions, which was not observed with the FOCE method; we therefore used the latter approach in the present study.

Two fitting procedures can be used [25, 35, 36]: maximum likelihood (ML) and restricted maximum likelihood (REML). The former was used to compare the models by goodness-of-fit statistics, given that it provides asymptotic efficient estimates, whereas the latter was used to obtain the final parameter estimates, as it yields unbiased estimates of variance components ([37], p. 746).

Model fitting was accomplished with the nlme function of the nlme package [38] of R statistical software [24].

### Calibration and subject-specific prediction

The mixed-effects modelling framework enables localization of the taper function to a new tree in a new plot with at least one additional diameter measurement. This process is known as calibration and involves prediction of random effects. Note that the calibration of a two-level mixed-effects model provides plot- and tree-level random effects, which can then be added to localize the function and yield SS predictions. According to the recommendations of [30], we used the FOCE method in the calibration and the SS prediction steps, to be consistent with the expansion method used in the fitting step. Numerous studies have mixed FO and FOCE methods (e.g. [20, 27, 39]): they used the former to predict random effects and the latter to make SS predictions, which may compromise the results obtained [30, 34]. For the FOCE method, the random effects for a plot (${\hat{b}}_{i}$) and their corresponding trees (${\hat{b}}_{ij}$) are aggregated in $\hat{b}$ and calculated as follows [36]:

$$\begin{array}{c} \hat{b}=\hat{D}Z_{i}^{{}^{\prime }}{\left(Z_{i}\hat{D}Z_{i}^{{}^{\prime }}+{\hat{R}}_{i}\right)}^{-1}\left[y_{i}-f\left(x_{i},\hat{\beta },\hat{b}\right)+Z_{i}\hat{b}\right] \end{array}$$ (2)

where

$$\begin{array}{c} \hat{b}=\left(\begin{array}{c} {\hat{b}}_{i1}\\ \vdots \\ {\hat{b}}_{in_{r}}\\ {\hat{b}}_{i11}\\ \vdots \\ {\hat{b}}_{i1n_{r}}\\ \vdots \\ {\hat{b}}_{in_{i}1}\\ \vdots \\ {\hat{b}}_{in_{i}n_{r}} \end{array}\right)\text{;}\;\hat{D}=\left(\begin{array}{cccc} {\hat{D}}_{p} & 0 & \cdots & 0\\ 0 & {\hat{D}}_{t1} & \cdots & 0\\ \vdots & \vdots & \ddots & \vdots \\ 0 & 0 & 0 & {\hat{D}}_{tn_{i}} \end{array}\right)\text{;}\;Z_{i}=\left(\begin{array}{ccccc} Z_{i1} & Z_{i1} & 0 & \cdots & 0\\ Z_{i2} & 0 & Z_{i2} & \cdots & 0\\ \vdots & \vdots & \vdots & \ddots & \vdots \\ Z_{in_{i}} & 0 & 0 & \cdots & Z_{in_{i}} \end{array}\right) \end{array}$$

$$\begin{array}{c} Z_{ij}=\left(\begin{array}{cccc} Z_{ij11} & Z_{ij12} & \cdots & Z_{ij1n_{r}}\\ Z_{ij21} & Z_{ij22} & \cdots & Z_{ij2n_{r}}\\ \vdots & \vdots & \ddots & \vdots \\ Z_{ijn_{ij}1} & Z_{ij2n_{r}} & \cdots & Z_{ijn_{ij}n_{r}} \end{array}\right)\text{;}\;y_{i}=\left(\begin{array}{c} y_{i11}\\ \vdots \\ y_{i1n_{ij}}\\ \vdots \\ y_{in_{i}1}\\ \vdots \\ y_{in_{i}n_{ij}} \end{array}\right) \end{array}$$

$$\begin{array}{c} {\hat{R}}_{i}={\hat{\sigma }}^{2}gM_{i}\text{;}\;M_{i}=\left(\begin{array}{cccc} M_{i1} & 0 & \cdots & 0\\ 0 & M_{i2} & \cdots & 0\\ \vdots & \vdots & \ddots & \vdots \\ 0 & 0 & \cdots & M_{in_{i}} \end{array}\right) \end{array}$$

where *f*(⋅) is a nonlinear function (the taper function); $\hat{\beta }$ is the vector of estimates of fixed parameters (β); *i* index stands for the plot number (*i*: 1, …, *m*); *j* index represents the tree number within plot *i* (*j*: 1, …, *n*_*i*_); *k* index represents the number of additional measurement of tree *j* (*k*: 1, …, *n*_*ij*_); *n*_*r*_ is the number of parameters to expand with random effects; ${\hat{b}}_{i1}-{\hat{b}}_{in_{r}}$ (${\hat{b}}_{i}$) and ${\hat{b}}_{i11}-{\hat{b}}_{ijn_{r}}$ (${\hat{b}}_{ij}$) are the estimates of plot- and tree-level random effects respectively; ${\hat{D}}_{p}$ and ${\hat{D}}_{tj}$ (${\hat{D}}_{t1}={\hat{D}}_{t2}=\cdots ={\hat{D}}_{tn_{i}}$) are the estimates of variance-covariance matrices of plot- and tree-level random effects respectively; $Z_{ijkr}=\frac{\partial f(x_{ijk},,b)}{\partial b_{r}}{|}_{\hat{\beta },\hat{b}}$ is the partial derivative of *f* with respect to the random effect *b*_*r*_ (it represents the random effect of the *r*-parameter expanded, the same for plot and tree level); **x**_*ijk*_ is the predictor vector of observation *k* of tree *j*; *y*_*ijk*_ is the additional measurement *k* of tree *j* within plot *i*; ${\hat{R}}_{i}$ is an estimate of the error matrix; ${\hat{\sigma }}^{2}$ is the estimate of the error variance; *g* is a variance function (1 if the residuals are homoscedastic); and **M**_*ij*_ is an *n*_*ij*_ × *n*_*ij*_ matrix, which equals an identity matrix for non-correlated within-tree observations and to a CAR(1)-structured matrix if within-tree correlation exists. Note that the partial derivatives of **Z**_*i*_ are equivalent to the partial derivatives with respect to the fixed parameters, because **b** enters linearly in the parameter vector. Taking into account that $\hat{b}$ appears on both sides of Eq 2, the calibration process must be solved iteratively [32]. After obtaining $\hat{b}$, the function can be localized at plot level, or at tree level, by adding respectively plot-level or plot- and tree-level random effects to the corresponding fixed parameters.

Eq 2 explains a general case of taper function calibration based on the hierarchical structure of the data, allowing several trees to be used per plot and several additional measurements to be made per tree to estimate the random effects. To assess whether trees from the same plot should be used together to estimate the plot-level random effect in calibration, we evaluated the variability explained by the plot and tree levels. Additionally, we only considered one additional diameter measurement per tree for calibration, because the improvement achieved by including more measurements does not usually compensate the field sampling effort required [15, 26, 40].

All heights along the stem where crosscut sections were measured in the field were considered in assessing the best stem location for the additional diameter measurement. The calibration procedure was implemented in R ([24], see S1 Appendix).

### Assessment of model performance

In addition to mixed-effects modelling, two fixed-effects models were fitted from Eq 1 by OLS (hereafter referred to as FMOLS) and by generalized least squares (GLS; hereafter referred to as FMGLS), which enables the error variance to be modelled for both heteroscedasticity and autocorrelation. The nls and the gnls functions, the latter from the nlme package [38] of R [24], were used for this purpose.

Akaike’s information criterion (AIC) and Schwarz’s Bayesian information criterion (BIC) were used to compare the two fixed-effects models and the 45 candidate mixed-effects models. For the latter, these statistics were obtained by assuming non-correlated within-tree residuals, although the autoregressive error structure was then included when necessary, because inclusion of an autocorrelation structure artificially improves the goodness-of-fit statistics (e.g. [41]). We used the random effects obtained from fitting step of the candidate mixed-effects models (i.e. those obtained by calibration with all measurements of all trees of all plots) to obtain SS predictions at plot- and tree-level.

For the calibration step (using the evaluation data set), the candidate mixed-effects models were localized using one additional diameter measurement per tree taken at different calibration heights. SS predictions of diameter outside bark (*d*_*i*_) and total stem volume (*v*) were subsequently obtained. These predictions were compared with those yielded by the fixed-effects models (FMOLS and FMGLS). We also considered the predictions of the mixed-effects models: (i) using only the fixed parameters, known as mean (M) response; and (ii) computing mean predictions of the mixed-effects model over the distribution of random effects, known as population-averaged (PA) response (e.g. [12, 42]).

The following statistics were used to compare the predictive performance of the models:

$$\begin{array}{c} \text{RMSE}=\sqrt{\frac{\sum_{i=1}^{m}\sum_{j=1}^{n_{i}}\sum_{k=1}^{n_{ij}}{\left(y_{ijk}-{\hat{y}}_{ijk}\right)}^{2}}{n-p}} \end{array}$$ (3)

$$\begin{array}{c} \bar{e}\%=100\frac{\bar{e}}{\bar{y}} \end{array}$$ (4)

where RMSE is the root mean square error; *y*_*ijk*_ and ${\hat{y}}_{ijk}$ are the observed and predicted values of the variable considered, respectively; *n* is the number of observations; *p* is the number of parameters; $\bar{e}\%$ is the percentage mean prediction error; $\bar{e}$ is the mean prediction error, obtained as $\sum_{i=1}^{m}\sum_{j=1}^{n_{i}}\sum_{k=1}^{n_{ij}}\left(y_{ijk}-{\hat{y}}_{ijk}\right)/n$; and $\bar{y}$ is the mean of the observed values of the dependent variable considered.

### Influence of random effects in stem taper

One objective of this study was to analyse the appropriateness of the strategy proposed by [12] for evaluating the variation in stem taper with the variation in parameter values. This type of analysis was used for assessing which part of the stem curve is affected by each parameter and for considering a parameter expansion combination in the mixed-effects model to account for variations in specific parts of the stem. These authors (op. cit.) evaluated how one parameter varied at a time, and therefore they did not take into account the correlation between random effects when more than one parameter was expanded. Moreover, they based the analyses on arbitrary parameter variations relative to fixed-effects model estimates.

In the present study, we selected the best mixed-effects model with two parameters expanded with random effects and the two mixed-effects models in which the previous parameters were expanded separately. The former enables consideration of the combined variation in parameter values, as it considers correlation between random effects, whereas the latter two are useful for evaluation of the individual variation in parameter values.

The estimates of the distribution of random effects obtained in the fitting step were used to obtain several quantiles of parameters and to simulate the stem taper variation over the mean response (M) of each model. This approach also allowed us to mimic the real variation in parameters in terms of magnitude, which constitutes a new procedure for understanding how the stem shape prediction varies with the inclusion of random effects.

## Results

When fitting mixed-effects models, 43 of the 45 candidate models reached convergence. Of these 43 models, 13 were excluded from further analysis because the fixed part of at least one parameter expanded with random effects was not significant at the 95% confidence level. Of the remaining 30 candidate models, in 14 of them at least one fixed parameter was non-significant at the specified level; the models were subsequently refitted by excluding these parameters. A CAR(1) error structure was required to account for autocorrelation in 21 of these 30 candidate models. The remaining 9 models were combinations of random effects in *a*_0_, *a*_1_, or *a*_2_, together with *b*_2_, *b*_3_, or *b*_4_.

**Fig 2:**
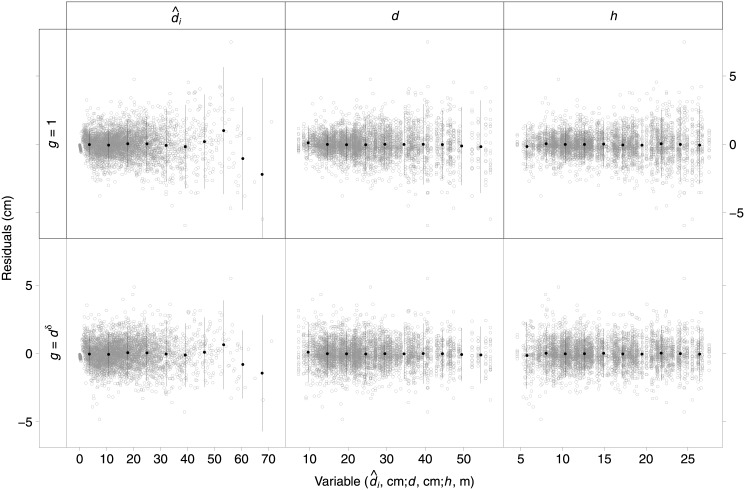
Standardized residuals of diameter outside bark against predicted values (left), diameter at breast height (middle) and total tree height (right), for a candidate mixed-effects model (*a*_1_ and *b*_3_ expanded with random effects), without (*g* = 1, top row) and with variance function (*g* = *d* ^*δ*^, bottom row). The black dots and vertical lines represent the means and confidence intervals of standardized residuals calculated for 10 intervals in which abscissa values were divided.

Heteroscedasticity was observed and accounted for with a variance function depending on the power of *d* (*g* = *d* ^*δ*^). Fig 2 shows standardized residuals of diameter outside bark (*d*_*i*_) against predicted values (${\hat{d}}_{i}$) and other covariates of stem taper function (*d* and *h*) for a candidate mixed-effects model (*a*_1_ and *b*_3_ expanded with random effects), without (*g* = 1) and with the variance function. The observed pattern of increase in residual variance with the value of the dependent variable predictions and independent variables (Fig 2, first row) disappeared after including the variance function (Fig 2, second row).

All candidate mixed-effects models in which one parameter was expanded with random effects performed worse than the models in which two parameters were expanded. The former were therefore not considered for further analysis. Three groups of mixed-effects models were subsequently defined, depending on which parameters included random effects and whether the CAR(1) structure was needed to model autocorrelation: (MM1) variable-exponent random-effects (all of the models within this group needed CAR(1)), (MM2) random effects both inside and outside the variable-exponent and including CAR(1), (MM3) random effects both inside and outside the variable-exponent without including CAR(1).

Table 2 shows the AIC and BIC values for the fixed-effects models (FMOLS and FMGLS) and for the best candidate mixed-effects models of each group in the fitting step. Moreover, the values of RMSE in *d*_*i*_ prediction were obtained for mean (FMOLS, FMGLS, and the M response of MM1-3) and SS responses for both plot and tree levels. The AIC and BIC values indicated that all of the candidate mixed-effects models (MM1-MM3) performed better than both the OLS and GLS fitting procedures. The RMSE values indicated that FMOLS slightly outperformed FMGLS and both performed better than M response of the three candidate mixed-effects models.

**Table 2:** AIC, BIC and RMSE (in diameter outside bark –*d*_*i*_, cm–, for mean, plot-level, and tree-level predictions) values for the fixed-effects models (fitted by OLS and GLS, first and second row, respectively) and for the best mixed-effects model of each group in the fitting step (*p* is the number of fixed parameters).

Name	Random	*p*	AIC	BIC	RMSE_*d*_*i*__		
					Mean	SS plot level	SS tree level
FMOLS	None	9	13042	13105	1.235	-	-
FMGLS	None	9	11476	11552	1.242	-	-
MM1	*b*_3_, *b*_6_	7	10097	10191	1.285	1.127	0.7552
MM2	*a*_1_, *b*_6_	8	10756	10856	1.323	1.186	0.8793
MM3	*a*_1_, *b*_3_	9	10790	10897	1.276	1.207	0.8272

The inclusion of the plot-level random effects in the mixed-effects models overcame the OLS response from 2 to 9%, while the inclusion of both plot- and tree-level random effects increased the accuracy by 33 to 39% relative to the OLS response. Therefore, we considered that calibration at plot level with several trees could be omitted, as the maximum predictive ability with the plot level alone is much lower than that of the tree level (both plot- and tree-level random effects). Thus, plot- and tree-level random effects were estimated for each additional stem diameter per tree in the evaluation data set and were used together to obtain SS predictions.

Table 3 shows the RMSE and percentage mean prediction error in predicted *d*_*i*_ and *v*, using fixed-effects models and the best candidate mixed-effects model from each group with the evaluation data set. In general, computing mean predictions from the mixed-effects models over the distribution of random effects (PA response) was more accurate than using the fixed part of mixed-effects models (M response). In addition, calibrating mixed-effects models increased their predictive ability relative to M and PA responses, and were also more accurate than fixed-effects models, except for MM2 and MM3 predictions of *d*_*i*_ and *v*, respectively.

**Table 3:** RMSE and percentage mean prediction error ($\hat{e}$, %) in diameter outside bark (*d*_*i*_, cm) and total tree volume (*v*, m^3^) predictions for the fixed-effects models (fitted by OLS and GLS, first and second row, respectively) and for the best mixed-effects model of each group in the fitting step considering: (i) considering only the fixed parameters (M), (ii) the mean predictions over the distribution of random effects (PA), and (iii) the random effects obtained from calibration using one additional diameter measurement per tree (SS).

Variable	Name	Random	RMSE			$\hat{e}$ (%)		
			M	PA	SS	M	PA	SS
*d*_*i*_	FMOLS	None	1.282	-	-	0.52	-	-
	FMGLS	None	1.305	-	-	0.58	-	-
	MM1	*b*_3_, *b*_6_	1.352	1.348	1.268	0.91	0.65	0.73
	MM2	*a*_1_, *b*_6_	1.355	1.358	1.316	−0.033	−0.17	0.097
	MM3	*a*_1_, *b*_3_	1.290	1.286	1.219	0.34	0.10	0.18
*v*	FMOLS	None	0.05676	-	-	1.8	-	-
	FMGLS	None	0.05872	-	-	2.2	-	-
	MM1	*b*_3_, *b*_6_	0.06083	0.05905	0.05624	2.9	2.3	2.0
	MM2	*a*_1_, *b*_6_	0.05742	0.05648	0.05175	1.3	0.86	0.79
	MM3	*a*_1_, *b*_3_	0.06557	0.06333	0.05714	2.3	1.6	1.4

**Fig 3:**
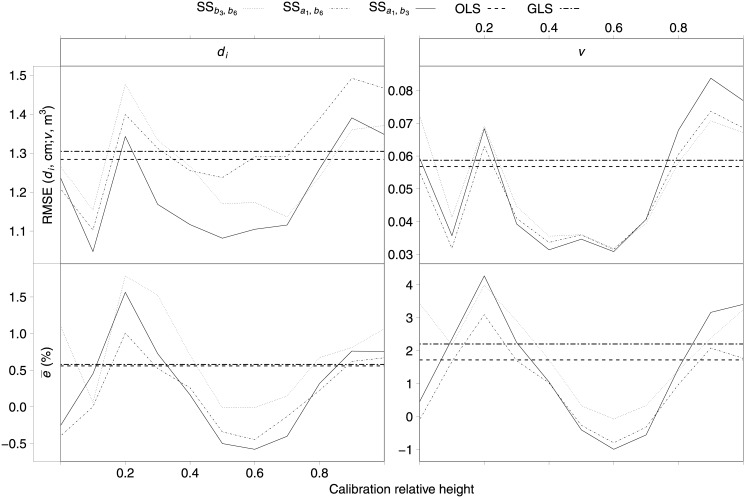
RMSE (top) and percentage mean prediction error (bottom) in diameter along the stem (*d*_*i*_, left) and total tree volume (*v*, right) predictions of the fixed-effects models (fitted by OLS and GLS techniques), and of the three candidate mixed-effects models (SS predictions obtained from calibration), disaggregated by calibration relative height classes.

Fig 3 shows the RMSE and percentage mean prediction error values obtained for SS response in predicting *d*_*i*_ and *v*, disaggregated by relative height classes at which the additional diameter for calibration was measured (hereafter referred to as calibration relative height classes). The OLS and GLS responses were included for comparative purposes. The SS predictions were less accurate and were slightly biased when the additional diameter for calibration was measured at the top or bottom of the tree. Calibration of the taper function using diameters measured between 40 and 60% of the total tree height improved the accuracy and reduced the bias relative to those yielded by the fixed-effects models (FMOLS and FMGLS). For these calibration relative height classes, the best mixed-effects model was the model that expanded the parameters *a*_1_ and *b*_3_ with random effects (MM3). Within the fixed-effects models, FMOLS showed better predictive ability than FMGLS.

**Fig 4:**
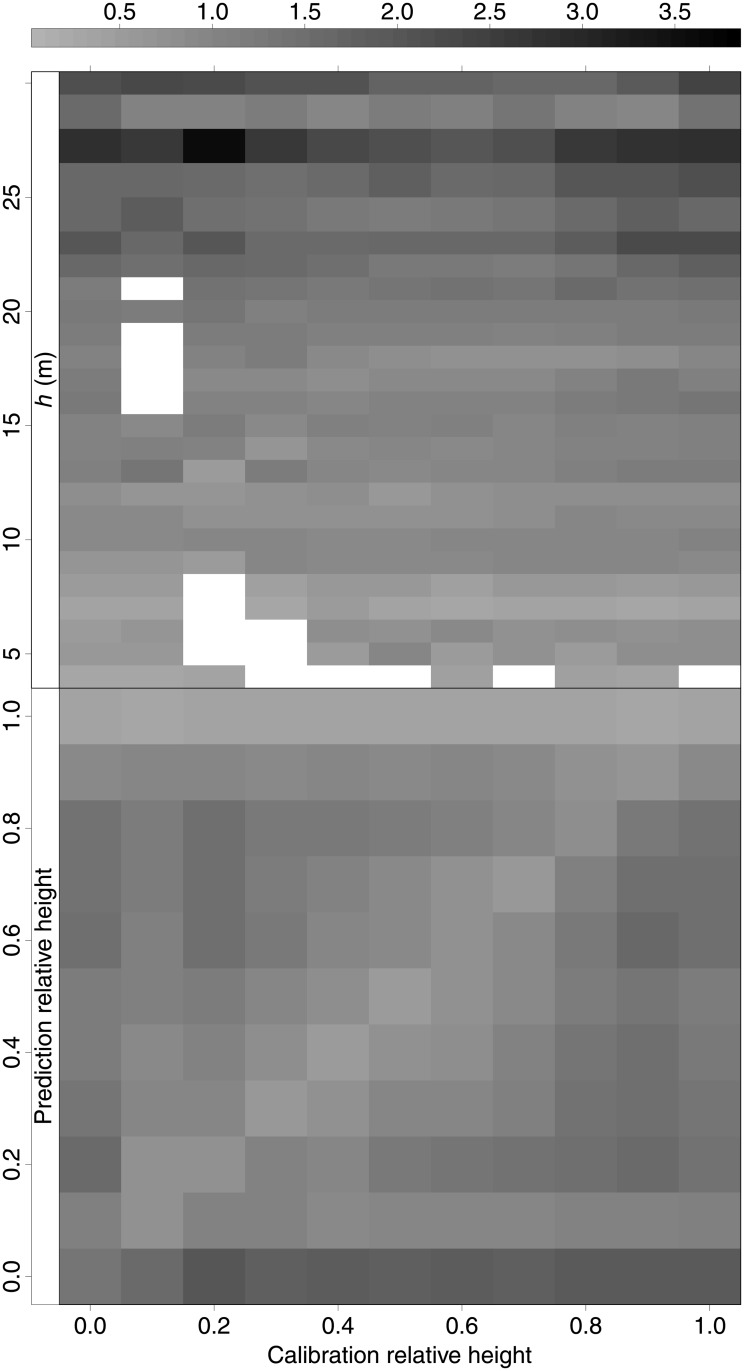
RMSE in SS predictions of diameter along the stem (*d*_*i*_, cm) for the mixed-effects model in which *a*_1_ and *b*_3_ were expanded with random effects, disaggregated by calibration relative height classes, total tree height (*h*, top), and prediction relative height classes (bottom).

The RMSE values of model MM3 in SS predictions for *d*_*i*_ were subsequently disaggregated by total tree height and prediction relative height classes (Fig 4). The top graph shows that RMSE values in *d*_*i*_ generally increased with total height of trees. The same pattern was found for OLS, GLS, M, and PA responses (graphs not shown). In addition, there was a lack of data for trees from 16 to 20 m in the 0.1 relative height class and for trees from 5 to 8 m in the 0.2 relative height class. We also observed that the lack of trees of 27 m in the evaluation data set (only one tree) caused an abnormally high RMSE in this range. Fig 4 (bottom) shows RMSE in *d*_*i*_ prediction by relative stem heights depending on the relative height at which the additional diameter for calibration was taken. As expected, use (for calibration) of diameters measured at the nearby parts of the stem section for which we predicted diameter yielded the lowest errors (see squares in the diagonal of Fig 4, bottom). Moreover, the highest RMSE values were obtained for basal tree log predictions, while the RMSE values were low for predictions at 10 and 100% of the total tree height, regardless of the relative height used for diameter measurement for calibration purposes. This general trend was also observed for OLS, GLS, M, and PA responses (graphs not shown).

**Fig 5:**
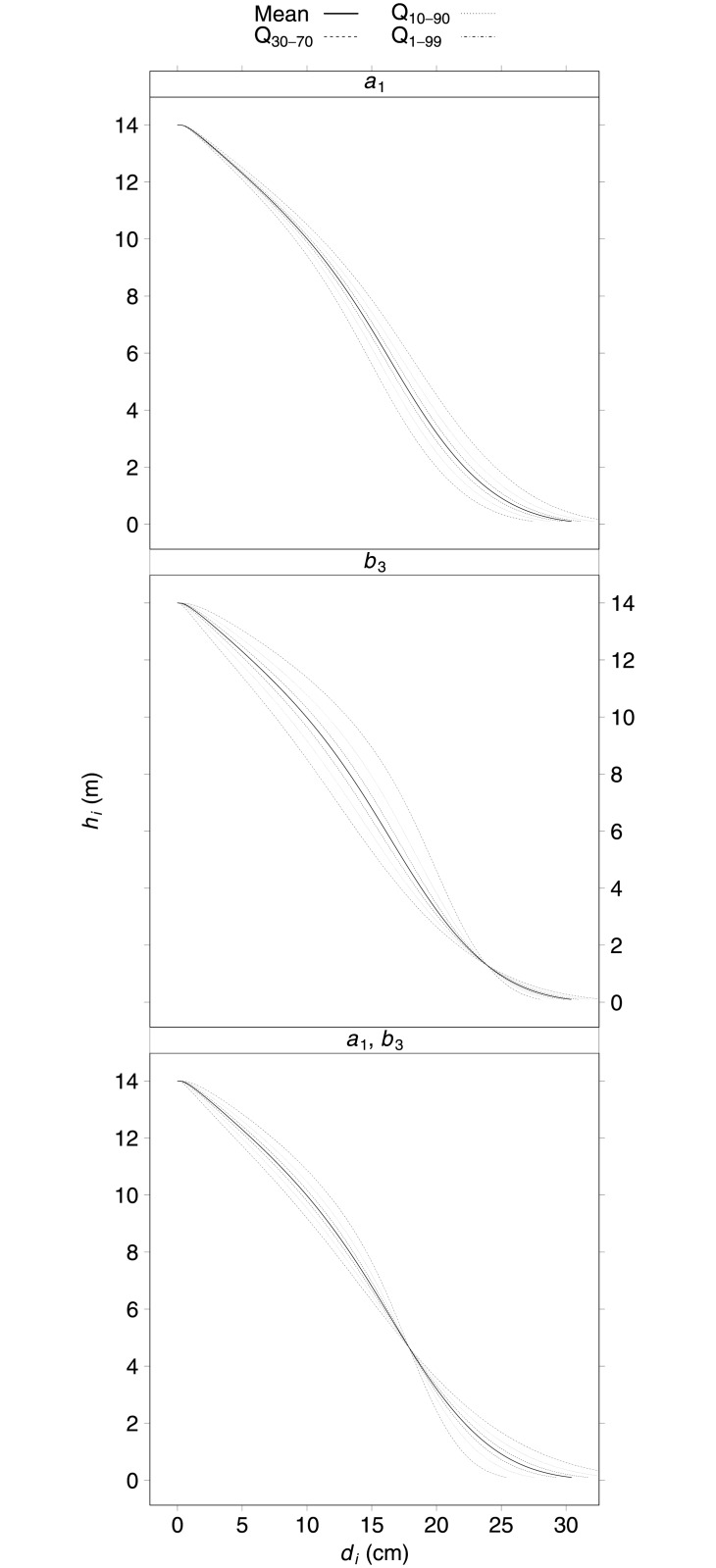
Variation of the stem taper (stem height against diameter along the stem, *h*_*i*_ and *d*_*i*_, respectively) for a tree with *d* = 24 cm and *h* = 14 m when varying *a*_1_ and *b*_3_ separately (top two) and jointly (bottom) for several quantiles (Q_30−70_, Q_10−90_, and Q_1−99_), obtained from the estimates of distribution of random effects for the corresponding mixed-effects model. The solid line corresponds to the M response.

Fig 5 (top two graphs) shows the variations in stem taper for a tree of *d* = 24 cm and *h* = 14 m, when parameters *a*_1_ and *b*_3_ vary separately. The variation in *a*_1_ affects the middle and bottom parts of the stem, whereas *b*_3_ variation affects the taper of the whole stem, constraining the curve to pass through the observed diameter at breast height, as the *x* value from Eq 1 is 1 when *h*_*i*_ equals 1.3 m. The lower graph in Fig 5 was obtained from the joint variation of *a*_1_ and *b*_3_. In this case, the model is forced to provide the same *d*_*i*_ value at approximately one-third of total tree height (4.7 m), regardless of the random effects values, and the stem curve is mainly modified in the lower and upper parts of the tree.

## Discussion

In the present study, random effects alone accounted for within-tree residual correlation. Using the same base model, [13] reported that mixed-effects models accounted for only part of the serial correlation, whereas [26] indicated that the correlation could be completely overcome by including random effects.

The plot level of the mixed-effects models explained much less variability than the tree level (see Table 2). Nevertheless, the plot level should not be omitted in the mixed-effects model as done in other studies (e.g. [13, 15, 20]). Moreover, we applied a likelihood-ratio test to the best model (*a*_1_ and *b*_3_ expanded with random effects) comparing the model in which plot and tree level were considered in random effects and the model in which plot level was ignored. The former proved significantly better at a confidence level of 95% (AIC of 10790 against 10807 respectively), therefore indicating that the plot level should be considered. The relative importance of each level was found to differ in studies dealing with mixed-effects models including trees and plots as levels. [16] used a linear mixed-effects model to describe the stem taper of Scots pine in Finland, and found that random variation between plots was initially higher than the variation between trees in an overall model, but it was lower than that when different regions were considered in the model. [43] applied multilevel mixed-effects modelling to a linear stem taper model for stone pine in Spain, and found that the variability between plots was about nine times greater than the variability between trees within the same plot. [17] and [44] observed that plot level variance was at least twice that of the tree level when applying multilevel mixed-effects modelling to describe individual tree height growth of Norway spruce and tree basal area increment of aspen, respectively. More recently, [45] used a multilevel mixed-effects model to describe diameter growth for China-fir in Southeast China, and indicated that variability between trees was higher than the variability between plots. These examples together suggest that the proportion of variability explained by each level depends on the data set used or on the type of relation to be modelled.

According to Fig 3, the values of RMSE when calibrating with diameters measured at 10–20% of the total tree height were not expected *a priori*, as they decreased sharply for a calibration relative height of 0.1 and increased for 0.2, in this case indicating even lower accuracy than for the fixed-effects models (FMOLS and FMGLS). A more detailed analysis showed that this was due to the lack of bole section measurements between 1.3 and 3.3 m, which roughly corresponds to 0.1–0.2 relative height for the experimental data. Taking into account the trend in RMSE with total tree height (top graph of Fig 4), when the results for all total tree heights were averaged, the lack of data led to a reduction in RMSE for the 0.1 calibration relative height class and an increase for the 0.2 class. Therefore, we excluded these stem parts for evaluation of the best height at which to measure the additional diameter for calibration.

When results from all available calibration heights were averaged, some candidate mixed-effects models yielded less accurate subject-specific predictions than the fixed-effects models. The poor performance is explained by the fact that calibration with additional diameters taken at the top or bottom of the tree decreased the performance of the mixed-effects model (Fig 3). This result suggests that these parts of the stem are not useful for explaining the variation in stem taper between plots and trees. In contrast, SS predictions clearly outperformed the predictive ability of fixed-effects models when the diameter for calibration was measured at between 40 and 60% of the total tree height. These height ranges are consistent with those proposed in some recent studies to improve taper function accuracy: [20] suggested a 60% value both for loblolly pine in southern United States and radiata pine in New Zealand, while [42] recommended measuring an additional diameter at 50% of total tree height for radiata pine in Spain. They are also consistent with those used as the starting point in other studies (50% [46, 47]).

Within the 40–60% relative height range, model MM3 (parameters *a*_1_ and *b*_3_ expanded with random effects) showed the best predictive ability. Note that MM3 was not the best model at the fitting step, which suggests that the smaller number of parameters in models MM1 and MM2 may have enhanced the corresponding goodness-of-fit statistics (Table 2). The mixed-effects model in which parameters *a*_1_ and *b*_3_ were expanded with random effects was refitted for the whole data set (both fitting and evaluation data sets) and the parameter estimates are shown in Table 4.

**Table 4:** Parameter estimates of the fixed-effects model (fitted by OLS, FMOLS) and the recommended mixed-effects model (expanding *a*_1_ and *b*_3_ with random effects, MM3), fitted using the whole data set.

Parameter	FMOLS	MM3 (*a*_1_, *b*_3_)
*a*_0_	0.9891	1.050
*a*_1_	0.9633	0.9427
*a*_2_	0.04585	0.04734
*b*_1_	0.3672	0.3619
*b*_2_	−0.3350	−0.6907
*b*_3_	0.5192	0.5847
*b*_4_	0.8471	1.126
*b*_5_	0.01777	0.02271
*b*_6_	−0.02647	−0.05812
$\sigma_{i,a_{1}}^{2}$		1.263 10^−5^
$\sigma_{i,b_{3}}^{2}$		8.273 10^−4^
*σ*_*i*, *a*_1_, *b*_3__		−1.104 10^−5^
$\sigma_{ij,a_{1}}^{2}$		1.205 10^−4^
$\sigma_{ij,b_{3}}^{2}$		3.095 10^−3^
*σ*_*ij*, *a*_1_, *b*_3__		3.847 10^−5^
*σ* ^2^	1.555	6.117 10^−3^
*δ*		0.7405

$\sigma_{i,a_{1}}^{2}$, $\sigma_{i,b_{3}}^{2}$ and *σ*_*i*, *a*_1_, *b*_3__, variances and covariance of random effects in parameters *a*_1_ and *b*_3_ at plot level; $\sigma_{ij,a_{1}}^{2}$, $\sigma_{ij,b_{3}}^{2}$ and *σ*_*ij*, *a*_1_, *b*_3__, variances and covariance of random effects in parameters *a*_1_ and *b*_3_ at tree level; *σ* ^2^, residual variance; *δ* parameter of power function. Note that the *σ* ^2^ of the mixed-effects model must be multiplied by *g* = *d* ^*δ*^ when applied (variance obtained from ordinary residuals is 0.6866).

In this study, the FOCE method was used consistently both in fitting and calibration phases. This method proved superior to the FO method in studies comparing these methods [30, 34], although the superiority is lower for models developed on the basis of subjects with few observations and high variability between subjects [34]. For these cases, other common methods can be used to estimate the likelihood function ([25], p. 312; [48]): the Laplacian approximation (e.g. [49]), the adaptative Gaussian quadrature rule (e.g. [50]) or Bayesian estimation (e.g. Markov Chain Monte Carlo –MCMC– integration, [49]).

Four alternatives were considered in this study for cases where no additional diameter is available for calibration: OLS and GLS can be used under the fixed-effects modelling approach, while the M and PA response can be obtained from the mixed-effects modelling approach. From the point of view of prediction (i.e. based on results from evaluation data set), the fixed-effects model fitted by OLS yielded the highest degree of accuracy, with negligible bias (see Table 3), although it violates the assumption of homocedasticity and the independence of within-tree observations. FMGLS accounted for these problems, but decreased the predictive performance of the model. On the other hand, M and PA responses were generally less accurate than the fixed-effects models (see Table 3 and Fig 3). These results are consistent with those reported in other studies (e.g. [42, 51]). Within mixed-effects modelling, PA response yielded generally better results than the M response, as also been reported in previous studies [12, 42], confirming that within the nonlinear mixed-effects modelling approach, the M response does not fully represent PA, because random effects enter in a nonlinear fashion [52]. Based on these results, and only for prediction purposes, we recommend use of the fixed-effects model fitted by OLS when no additional diameter for calibration is available. As done for the best candidate mixed-effects model, the base model was refitted by OLS for the whole data (Table 4).

Regarding disaggregation of RMSE values for SS response in *d*_*i*_ predictions by prediction of relative heights (Fig 4, bottom), we observed that the improvement in the predictive ability mainly focuses on the part of the bole around the diameter measurement used for calibration, which was also pointed out by [40]. This is logical because the stem curve is modified to pass close to the additional diameter used. The small errors observed for 10 and 100% of total tree height are explained by the fact that these relative heights correspond to breast height and total tree height, respectively, and the [7] equation returns zero diameter for *h*_*i*_ = *h* and passes close to diameter at breast height when *h*_*i*_ = 1.3 m.

Basal log appears to be the most difficult-to-predict part of the stem (see Fig 4, bottom), except when an additional diameter from this part is used for calibration (bottom left square of Fig 4, bottom). Therefore, if the main interest is accurate prediction of the shape of basal stem log rather than the whole stem, we recommend calibrating the mixed-effects model by measuring the extra diameter at this part of the stem.

Fig 5 demonstrates that the variation in stem taper varies depending on how parameter values vary. As we hypothesized, stem taper may differ substantially depending on whether or not the correlation between random effects is taken into account, which can lead to incorrect assessment of the sensitivity of stem taper to parameter variations. Within this context, we demonstrated that the approach of varying one parameter at a time, proposed by [12], is only valid when just one parameter is expanded. In this case, this information could be used to indicate about which parameter should be expanded with random effects. Otherwise, fitting several candidate mixed-effects models by expanding different parameters with random effects should be considered.

## Conclusions

A nonlinear mixed-effects stem profile model was developed for maritime pine stands in NW Spain on the basis of the variable-exponent taper function of [7]. This study represents the first application of multilevel mixed-effects modelling approach to nonlinear stem taper functions. In the fitting step, tree level accounted for much more variability than the plot level in multilevel mixed-effects models. Therefore, subject-specific predictions were obtained with the joint use of plot- and tree-level random effects.

The calibration process generally improved the predictions of the fixed-effects models fitted by OLS and GLS and those of the fixed part of the mixed-effects models (M response) and the mean predictions of mixed-effects models over the distribution of random effects (PA response). Expanding the parameters *a*_1_ and *b*_3_ with random effects and taking the additional stem diameter for calibration from 40 to 60% of the total tree height yielded the most accurate SS predictions of diameter outside bark along the stem (*d*_*i*_) and total tree volume (*v*). For practical application, when no additional diameter is available for calibration, we recommend use of the fixed-effects model fitted by OLS.

In mixed-effects modelling, when deciding which parameters must be expanded with random effects according to the parts of the stem they influence, the option of varying one parameter at a time can only be considered when one parameter is expanded with random effects. Otherwise, correlation between random effects must be taken into account by fitting a mixed-effects model.

## Supporting Information

S1 Appendix R implementation of the calibration procedure for a multilevel mixed-effects model based on stem taper function of Kozak (2004). (ZIP)

S1 Dataset Stem measurements of 420 trees of *Pinus pinaster* Ait. from Asturias. (ZIP)
