# Correction: Fitting and Calibrating a Multilevel Mixed-Effects Stem Taper Model for Maritime Pine in NW Spain

**DOI:** 10.1371/journal.pone.0151297

**Published:** 2016-03-03

**Authors:** Manuel Arias-Rodil, Fernando Castedo-Dorado, Asunción Cámara-Obregón, Ulises Diéguez-Aranda

In Table 4, the value for δ in the “MM3 (*a*_1_, *b*_3_)” column is incorrect. Please see the corrected Table 4 here.

**Table 4:** Parameter estimates of the fixed-effects model (fitted by OLS, FMOLS) and the recommended mixed-effects model (expanding *a*_1_ and *b*_3_ with random effects, MM3), fitted using the whole data set.

Parameter	FMOLS	MM3 (*a*_1_, *b*_3_)
*a*_0_	0.9891	1.050
*a*_1_	0.9633	0.9427
*a*_2_	0.04585	0.04734
*b*_1_	0.3672	0.3619
*b*_2_	-0.3350	-0.6907
*b*_3_	0.5192	0.5847
*b*_4_	0.8471	1.126
*b*_5_	0.01777	0.02271
*b*_6_	-0.02647	-0.05812
$\sigma_{i{,}_{a_{1}}}^{\text{2}}$	σ^2^_i_,	1.263 10^−5^
$\sigma_{i{,}_{b}{}_{3}}^{\text{2}}$		8.273 10^−4^
$\sigma_{i{,}_{a_{1}}{,}_{b_{3}}}^{\text{2}}$		−1.104 10^−5^
$\sigma_{ij{,}_{a_{1}}}^{2}$		1.205 10^−4^
$\sigma_{ij{,}_{b_{3}}}^{2}$		3.095 10^−3^
$\sigma_{ij{,}_{a_{1}}{,}_{b_{3}}}^{2}$		3.847 10^−5^
σ^2^	1.555	6.117 10^−3^
δ		1.481

$\sigma_{i{,}_{a_{1}}}^{\text{2}}$,$\sigma_{i{,}_{b}{}_{3}}^{\text{2}}$ and $\sigma_{i{,}_{a_{1}}{,}_{b_{3}}}^{\text{2}}$, variances and covariance of random effects in parameters *a*_1_ and *b*_3_ at plot level; $\sigma_{ij{,}_{a_{1}}}^{2}$,$\sigma_{ij{,}_{b_{3}}}^{2}$ and $\sigma_{ij{,}_{a_{1}}{,}_{b_{3}}}^{2}$, variances and covariance of random effects in parameters *a*_1_ and *b*_3_ at tree level; σ^2^, residual variance; δ parameter of power function. Note that the σ^2^ of the mixed-effects model must be multiplied by *g* = *d*^*δ*^ when applied (variance obtained from ordinary residuals is 0.6866).

The value for “delta” in the S1 Appendix is incorrect. Please view the correct S1 Appendix below.

## Supporting Information

S1 Appendix R implementation of the calibration procedure for a multilevel mixed-effects model based on stem taper function of Kozak (2004). (ZIP)
