# Supplementary material for: Fitting and Calibrating a Multilevel Mixed-Effects Stem Taper Model for Maritime Pine in NW Spain
Source: PLoS One. 2015 Dec 2;10(12):e0143521. doi: 10.1371/journal.pone.0143521 (PMC4668033; doi:10.1371/journal.pone.0143521)
Supplement: S1 Appendix — (ZIP) [file pone.0143521.s001.zip › S1Appendix.pdf]

S1 Appendix

R implementation of the calibration procedure for a multilevel mixed-effects model based on the stem taper function of Kozak (2004)

This Appendix shows the implemetation in R [1] of the calibration procedure for the multilevel mixed-effects model based on the Kozak [2] equation. The function that estimates random effects (`EstimateRandomEffects`) uses several arguments: (i) a plot-level `data.frame` with the additional measurements for calibration (`dfplot`), (ii) a names vector of parameters to expand with random effects (`randparms`), (iii) estimates from the fitting step (fixed parameters, `fparms`; variance-covariance matrices of plot- and tree-level random effects, `Dp` and `Dt`, respectively; residual variance, `sigma2`; and δ value to use for weighting residuals, `delta`), and (iv) relative tolerance criteria to end the calibration procedure (`tolerance`). This function is a general implementation of the calibration procedure, which in our case uses the Kozak [2] stem taper function (`ComputeDiKozak`). Thus, although it can be used for the same stem taper function but by expanding different parameters with random effects, the code can also be easily adapted to other stem taper functions, or even other type of models, with few changes required.

In the present study, random variation in stem shape was low between plots, and we therefore considered the joint estimation of plot- and tree-level random effects for each individual tree (and using one observation per tree). However, the `EstimateRandomEffects` function allows random effects estimates to be obtained by using information on more than one tree per plot and more than one observation per tree: this was exemplified when applying the function over `dfplot.threetrees`.

```
# 1.Authorship ----------------------------------------------------------
# Manuel Arias-Rodil and Ulises Diéguez-Aranda
# 2015

# 2.File description ----------------------------------------------------
# S1 Appendix. R implementation of the calibration procedure for a multilevel mixed-
    effects model based on stem taper function of Kozak (2004)
# Kozak, A. (2004). My last words on taper equations. The Forestry Chronicle, 80(4),
```

507515.

```
# Arguments passed to functions
# hi: height along the stem (m)
# h: total tree height (m)
# d: diameter at breast height (cm)
# parms: parameter vector of Kozak (2004) equation
# b: random effects
# randparms: parameter names in which random effects will be considered
# parmnames: parameter names
# dfplot: data.frame with additional information for calibration
#         tree: number of tree
#         h: total tree height (m)
#         d: diameter at breast height (cm)
#         hi: height along the stem (m)
#         di: diameter along the stem (cm)
# fparms: fixed-effects parameter vector
# Dp: variance-covariance matrix for plot-level random effects
# Dt: variance-covariance matrix for tree-level random effects (within plot)
# sigma2: residual variance
# delta: Delta value for residual weights (g = var.e * d ^ delta)
# tolerance: relative tolerance criteria for random-effects estimation

# 3.library() statements ---------------------------------------------------
# Load library
library(Matrix)

# 4.Function definitions ---------------------------------------------------
# Function to compute diameter along the stem using the Kozak (2004) model, expanding a1
  and b3 with random effects
ComputeDiKozak <- function(hi, h, d, parms, b, randparms, parmnames){
  rp <- parmnames %in% randparms
  bp <- bt <- rep(0, length(parmnames))
  bp[which(rp)] <- b[1:length(randparms)]  # Plot-level random effects
  bt[which(rp)] <- b[(length(randparms) + 1):(2 * length(randparms))]  # Tree-level
    random effects
  prms <- parms + rp * bp + rp * bt
  p <- 1.3 / h
  Qi <- 1 - (hi / h) ^ (1 / 3)
```

```
  Xi <- (Qi) / (1 - p ^ (1 / 3))
  zi <- hi / h
  di <- prms[["a0"]] * d ^ prms[["a1"]] * h ^ prms[["a2"]] * Xi ^ (prms[["b1"]] * zi ^ 4
      + prms["b2"] * (1 / exp(d / h)) + prms[["b3"]] * Xi ^ 0.1 + prms[["b4"]] * (1 / d)
      + prms[["b5"]] * h ^ Qi + prms[["b6"]] * Xi)
  return(di)
}

# Function to estimate random parameters for both plot and tree levels
EstimateRandomEffects <- function(dfplot, randparms, fparms, Dp, Dt, sigma2, delta,
    tolerance = 1e-4){
  nrand <- length(randparms)
  treename <- unique(dfplot$tree)
  ntree <- length(treename)
  nobs <- nrow(dfplot)
  postree <- tapply(1:nobs, dfplot$tree, min)
  ltree <- tapply(dfplot$tree, dfplot$tree, length)
  lrand <- nrand + nrand * ntree
  rp <- names(fparms) %in% randparms
  # D matrix
  Dlist <- list()
  Dlist[[1]] <- Dp
  for(i in 2:(ntree + 1)){
    Dlist[[i]] <- Dt
  }
  D <- as.matrix(bdiag(Dlist))
  # Ri matrix
  Mi <- diag(1, nrow = nobs, ncol = nobs)
  if(!missing(delta)){  # Weighting structure, if needed
    Mi.list <- list()
    for(i in 1:ntree){
      M.ij <- Mi[postree[i]:(postree[i] + ltree[i] - 1), postree[i]:(postree[i] + ltree[i
          ] - 1)]
      d.ij <- dfplot$d[postree[i]]
      Mi.list[[i]] <- M.ij * d.ij ^ (2 * delta)  # g function
    }
    Mi <- as.matrix(bdiag(Mi.list))
  }
  Ri <- sigma2 * Mi
```

```
# Additional diameter/s used for calibration
yi <- dfplot$di # Diameters along the stem
# Initial values for iterative procedure to estimate random effects
b.0 <- rep(0, lrand)
tol <- rep(1, lrand)
# Iterative procedure ("while" loop)
while (sum(tol > tolerance) > 0){ # Stop if all tol values are bigger than 1e-4
  # Z matrix and f(x_i, B, b)
  Zlist <- list()
  fxiBblist <- list()
  posrand <- seq(nrand + 1, lrand, nrand)
  for(i in 1:ntree){
    posrand.i <- posrand[i]
    b.z <- c(b.0[1:nrand], b.0[posrand.i:(posrand.i + nrand - 1)])
    tree.obs <- dfplot[postree[i]:(postree[i] + ltree[i] - 1), ]
    Zij <- attr(numericDeriv(quote(ComputeDiKozak(hi = tree.obs$hi, h = tree.obs$h, d =
         tree.obs$d, parms = fparms, b = b.z, randparms = randparms, parmnames = names(
        fparms))), theta = "b.z"), "gradient")[, (nrand + 1):(2 * nrand)]
    fxiBb <- ComputeDiKozak(hi = tree.obs$hi, h = tree.obs$h, d = tree.obs$d, parms =
        fparms, b = b.z, randparms = randparms, parmnames = names(fparms))
    if(is.null(nrow(Zij))) Zij <- matrix(Zij, nrow = 1)
    Zlist[[i]] <- Zij
    fxiBblist[[i]] <- fxiBb
  }
  Zi.plot <- do.call(rbind, Zlist)
  Zij.trees <- as.matrix(bdiag(Zlist))
  Zi <- cbind(Zi.plot, Zij.trees)
  fxiBb <- do.call(c, fxiBblist)
  # Random-effects estimation (Lindstrom & Bates, 1990; First Order Conditional
      Expectation method, FOCE)
  b <- D %*% t(Zi) %*% solve (Ri + Zi %*% D %*% t(Zi)) %*% ((yi - fxiBb) + Zi %*% b.0)
  if (all(b.0 == 0)) b.prev <- rep(1, lrand) else b.prev <- b.0
  tol <- abs((b - b.prev) / b.prev)  # Compute relative difference with random effects
      of previous iterations
  b.0 <- b
}
bi <- split(b, ceiling(seq_along(b) / nrand), )
names(bi) <- c("Plot", paste("Tree", treename, sep = ""))
bi <- do.call(rbind, bi)
```

```
  colnames(bi) <- randparms
  return(bi)
}

# 5.Execution statements --------------------------------------------------
# Fixed-effects parameters of fixed-effects model fitted with OLS and mixed-effects model
fparms.fmols <- c(a0 = 0.9891, a1 = 0.9633, a2 = 0.04585, b1 = 0.3672, b2 = -0.3350, b3 =
     0.5192, b4 = 0.8471, b5 = 0.01777, b6 = -0.02647)
fparms.mm3 <- c(a0 = 1.050, a1 = 0.9427, a2 = 0.04734, b1 = 0.3619, b2 = -0.6907, b3 =
    0.5847, b4 = 1.126, b5 = 0.02271, b6 = -0.05812)

# Variance-covariance estimates of random effects both for plot and tree levels
Dp <- matrix(c(1.263e-5, -1.104e-5, -1.104e-5, 8.273e-4), nrow = 2, byrow = T)
Dt <- matrix(c(1.205e-4, 3.847e-5, 3.847e-5, 3.095e-3), nrow = 2, byrow = T)

# Residual variance
sigma2 <- 6.117e-03

# Delta value for residual weights (g = sigma2 * d ^ delta)
delta <- 0.7405

# Calibration with one tree per plot (as done in the present study)
dfplot.onetree <- data.frame(tree = 1, h = 16.02, d = 32.7, hi = 9.27, di = 16.2)
EstimateRandomEffects(dfplot = dfplot.onetree, randparms = c("a1", "b3"), fparms = fparms
    .mm3, Dp = Dp, Dt = Dt, sigma2 = sigma2, delta = delta)

# Calibration with more than one tree per plot and more than one observation per tree
dfplot.threetrees <- data.frame(tree = c(1, 2, 2, 3),
                                h = c(16.02, 13.23, 13.23, 12.88),
                                d = c(32.7, 26.3, 26.3, 21.3),
                                hi = c(9.27, 5.43, 9.34, 6.46),
                                di = c(16.2, 14.85, 8.65, 10.64))
EstimateRandomEffects(dfplot = dfplot.threetrees, randparms = c("a1", "b3"), fparms =
    fparms.mm3, Dp = Dp, Dt = Dt, sigma2 = sigma2, delta = delta)
```

References

[1] R Core Team. *R: a language and environment for statistical computing.* Vienna, Austria, 2015.

[2] Kozak, A. My last words on taper equations. *For. Chron.* 2004; 80(4): 507–515.
