# Supplementary material for: Fitting and Calibrating a Multilevel Mixed-Effects Stem Taper Model for Maritime Pine in NW Spain
Source: PLoS One. 2015 Dec 2;10(12):e0143521. doi: 10.1371/journal.pone.0143521 (PMC4668033; doi:10.1371/journal.pone.0143521)
Supplement: S1 Dataset — (ZIP) [file pone.0143521.s002.zip › S1Dataset.pdf]

S1 Dataset

Stem measurements of 420 trees of *Pinus pinaster* Ait. from Asturias

The dataset comprises stem measurements taken from 420 trees of *Pinus pinaster* Ait. from Asturias, and includes the following variables:

- `dataset`: this variable indicates whether the observation belongs to fitting or evaluation data set
- `plot`: plot number
- `tree`: tree number
- `d`: diameter at breast height (cm)
- `h`: total tree height (m)
- `hst`: stump height (m)
- `hi`: stem height (m)
- `di`: stem diameter (cm)
- `vi`: log volume (m^3)
- `cumvi`: cumulative tree volume (m^3) from stump to `hi`
- `v`: total tree volume (m^3)
